# Organic micropollutants paracetamol and ibuprofen—toxicity, biodegradation, and genetic background of their utilization by bacteria

**DOI:** 10.1007/s11356-018-2517-x

**Published:** 2018-06-19

**Authors:** Joanna Żur, Artur Piński, Ariel Marchlewicz, Katarzyna Hupert-Kocurek, Danuta Wojcieszyńska, Urszula Guzik

**Affiliations:** 1 Department of Biochemistry, Faculty of Biology and Environmental Protection, University of Silesia in Katowice, Jagiellońska 28, 40-032 Katowice, Poland; 2 Department of Plant Anatomy and Cytology, Faculty of Biology and Environmental Protection, University of Silesia in Katowice, Jagiellońska 28, 40-032 Katowice, Poland

**Keywords:** Paracetamol, Ibuprofen, Monocyclic NSAIDs, Biodegradation, Biotransformation, Gene clusters

## Abstract

Currently, analgesics and nonsteroidal anti-inflammatory drugs (NSAIDs) are classified as one of the most emerging group of xenobiotics and have been detected in various natural matrices. Among them, monocyclic paracetamol and ibuprofen, widely used to treat mild and moderate pain are the most popular. Since long-term adverse effects of these xenobiotics and their biological and pharmacokinetic activity especially at environmentally relevant concentrations are better understood, degradation of such contaminants has become a major concern. Moreover, to date, conventional wastewater treatment plants (WWTPs) are not fully adapted to remove that kind of micropollutants. Bioremediation processes, which utilize bacterial strains with increased degradation abilities, seem to be a promising alternative to the chemical methods used so far. Nevertheless, despite the wide prevalence of paracetamol and ibuprofen in the environment, toxicity and mechanism of their microbial degradation as well as genetic background of these processes remain not fully characterized. In this review, we described the current state of knowledge about toxicity and biodegradation mechanisms of paracetamol and ibuprofen and provided bioinformatics analysis concerning the genetic bases of these xenobiotics decomposition.

## Introduction

A wide range of unique emerging pollutants, including pharmaceuticals and their metabolites or residues, are continuously introduced into various environments mainly from hospital and municipal wastewater, consumer use, or disposal and discharges from pharmaceutical production (Hu et al. 2013; Zhang et al. 2013; Ebele et al. 2017). Drugs that reach the environment are mainly excreted in unmodified or slightly metabolized form, i.e., hydroxylated or conjugated with charged molecules such as glutathione, glucuronic acid, or sulfate (Marchlewicz et al. 2015). To date, wastewater treatment plants (WWTPs) are not designed to completely remove this kind of organic micropollutants, often characterized by high water solubility and poor biodegradability, which favor their environmental persistence (Candido et al. 2017). Moreover, conventional treatment processes, e.g., chlorination usually results in formation of highly toxic intermediates (Cao et al. 2016). Nonetheless, for some active compounds, sewage treatment may eliminate up to 99.9% of their initial concentration (Chonova et al. 2017). Due to the extremely diversified structure of micropollutants, their unsaturated/saturated character, the presence of various functional groups (e.g., halogen, sulfate), different side chains, and linear or branched structure, there is no one specific method used in the sewage treatment for micropollutants utilization. However, some advanced treatment technologies, enabling a high removal rate of micropollutants should be highlighted. Currently, the most important techniques are adsorption on a powdered activated carbon (PAC) and granular activated carbon (GAC), biofiltration including a trickling filter, sand filtration processes, and biological activated carbon (BAC), nanofiltration, reverse osmosis, membrane bioreactors, attached growth technology, and carbon nanocomposites with magnetic properties ((AC)/CoFe_2_O_4_) (Luo et al. 2014; Saucier et al. 2017). Lack of the optimal treatment technique leads to the release of many pollutants in their parent form or as active or more stable metabolites to the environment (Rozas et al. 2016). This could results in their accumulation in trophic chains and/or long-term adverse effects on aquatic organism (Grujíć et al. 2009). Some authors highlight that environmentally relevant concentrations (ng/L to μg/L) of residual pharmaceuticals may not induce negative effects. Although literature data regarding non-target organism receptors sensitivity, e.g., primary producers, cnidarians, cladocerans, mussels, or fish, are scarce, due to the mechanism of pharmaceutical action, they can presumably pose a risk for these organisms (Parolini and Binelli 2012). Oliveira et al. (2015) pointed out some features of drugs, which may promote their negative influence on biocoenosis, e.g., high lipophilic character, which favors the bioaccumulation, low biodegradability, pseudopersistence, since the rate of pharmaceuticals removal is compensated by the daily input of new molecules into the environment. Additionally, drugs are designed as biologically active molecules, which should be effective in a number of different species in a slowly metabolized form and their residues may interact with other pharmaceuticals in natural matrices. Nevertheless, Nunes et al. (2015) noticed that due to the conservative nature of the physiological processes and similarity of target molecules in humans and many aquatic organisms, some consequences of the continuous contamination of the environment with pharmaceuticals at the population and ecosystem levels are possible to predict. Parolini and Binelli (2012) warned that high activity of pharmaceuticals can seriously affect the health status of the biocoenosis. Moreover, Elersek et al. (2016) noticed that even if individual concentration of drug is low, the presence of other pharmaceuticals with common mode of action might induce additive or synergistic effects. Thus, in a simple way, environmental risk calculated on the basis of ecotoxicological data available for particular drug may result in underestimation of the actual toxic effect of micropollutants. Additionally, the choice of the most optimal model for calculation of toxicological effects, e.g., sigmoid-fitting, concentration addition or independent action can lead to different results (Elersek et al. 2016). One of the most prevalent anthropogenic group of xenobiotics, which have been detected in soil, sediments, and surface, ground, or even drinking water are analgesics and nonsteroidal anti-inflammatory drugs (NSAIDs). From the environmental point of view, annual production of NSAIDs, which oscillates around several kilotons, their increasing consumption and prevalence in medicine must be reflected in natural matrices (Parolini et al. 2011).

Monocyclic over-the-counter NSAIDs, such as ibuprofen, acetylsalicylic acid, and paracetamol, which share anti-inflammatory, analgesic, and antipyretic action, are the most popular and available drugs among this class. Since aggressive chemical treatment methods, mainly advanced oxidation processes (AOP) are characterized by harsh reaction conditions and are not adapted to remove analgesics, bioremediation processes utilizing bacterial strains with increased abilities to degrade xenobiotics seem to be a promising alternative. Effectiveness of selected advanced wastewater treatment methods used in analgesics and NSAIDs removal are summarized in Table 1.

**Table 1:** Selected advanced treatment methods used in paracetamol and ibuprofen removal

Compound	Treatment	Conditions	Removal effectiveness [%]	Reference
Ibuprofen	Coagulation-flocculation	FeCl_3_/Al_2_(SO_4_)_3_ Dosage: 25, 50 mg/L	12.0 ± 4.8	Suárez et al. (2009), Luo et al. (2014)
Ibuprofen	Ozonation/AOP	UV_254_, 10 min	34	Luo et al. (2014)
Ibuprofen	Ozonation/AOP	UV_254_ + H_2_O_2_ (50 mg/L); 10 min, 30 min	100 (10 min), 100 (30 min)	Luo et al. (2014)
Ibuprofen	Ozone oxidation	Initial concentration: 1 mg/L 160 mg/L for 20 min, pH 9, at 25 °C	99	Wang and Wang (2016)
Ibuprofen	Membrane processes	PES flat-sheet, 100 kDa; TMP = 0.5 ± 0.01 bar	7	Jermann et al. (2009), Luo et al. (2014)
Ibuprofen	Membrane processes	Filmtec TW30; TMP = 9.5–10.2 bar	> 99	Sahar et al. (2011), Yangali-Quintanilla et al. (2011), Luo et al. (2014)
Ibuprofen	Membrane bioreactor (MBR)	Full-scale HF a (Koch Puron); MAb 235 m^2^; pore size 0.1–0.2 μm	~ 100	Trinh et al. (2012), Luo et al. (2014)
Ibuprofen	Membrane bioreactor (MBR)	Lab-scale submerged HF UF module; MA 0.047 m^2^; pore size 0.04 μm; SRT: 70 days; HRT: 24 h; MLSS: 8.6–10 g/L	96.7 ± 0.7	Tadkaew et al. (2011), Luo et al. (2014)
Ibuprofen	Membrane bioreactor (MBR)	Lab-scale polyvinylidene fluoride HF; MA 0.2 m^2^; pore size 0.4 μm; HRT: 1 or 3 days; MLSS: 2.3–4.6 g/L	~ 100	Bo et al. (2009), Luo et al. (2014)
Ibuprofen	Attached growth treatment processes	Media: bioplastic-based biofilm carriers; volume: 2.5 L	~ 100	Falås et al. (2012), Luo et al. (2014)
Ibuprofen	Activated sludge with high nitrifying activity in sequencing batch reactor (SBR)	Biodegradation after 24 h in water	76	Kruglova et al. (2016)
Ibuprofen	Grit channels + primary clarifies + conventional activated sludge	Initial concentration: 4500 ng/L	99.7	Blair et al. (2015)
Ibuprofen	Primary treatment + Orbal oxidation ditch + UV disinfection	Initial concentration: 130–450 ng/L	60–90	Sun et al. (2014)
Ibuprofen	Fenton oxidation	Initial concentration: 0.87 mM Fenton, 30 °C, pH 3, 2 h, Fe_2_ + 25% 1.2 mM, H_2_O_2_ 25% 0.32 mM	> 50	Wang and Wang (2016)
Paracetamol	Activated sludge + disinfection	–	75	Kosma et al. (2010), Al Qarni et al. (2016)
Paracetamol	Activated sludge + sand filtration	–	95.6	Kosma et al. (2010), Al Qarni et al. (2016)
Paracetamol	Activated sludge + trickling filter	–	94	Kasprzyk-Hordern et al. (2009), Al Qarni et al. (2016)
Paracetamol	Activated sludge	–	> 99	Kasprzyk-Hordern et al. (2009), Al Qarni et al. (2016)
Paracetamol	Membrane bioreactor − ultrafiltration	–	> 99	Kovalova et al. (2012), Al Qarni et al. (2016)
Paracetamol	Membrane bioreactor	–	> 99	Nielsen et al. (2013), Al Qarni et al. (2016)
Paracetamol	Modified Bardenpho process	Initial concentration: 218,000 ng/L	99	Rajbongshi et al. (2016)
Paracetamol	Powdered activated carbon (PAC) 5 mg/L	Initial concentration: 100 ng/L	~ 70	Snyder et al. (2007), Wang and Wang 2016
Paracetamol	Graphene (0.1 g/L)	Initial concentration: 1 mg/L	46	Yang and Tang (2016), Wang and Wang (2016)
Paracetamol	Ozone oxidation	Initial concentration: 1 μM, 0–6.8 mM for 24 h, pH 7.2, 20 °C	100	Wang and Wang (2016)
Paracetamol	Fenton oxidation	Initial concentration: 100 mg/L 60 °C, pH 2.6, 5 h, magnetite 25% 6 g/L, H_2_O_2_ 25% 28 mM	100	Wang and Wang (2016)

*PES* polyethersulfone, *TMP* trans-membrane pressure, *HF* hollow fiber, *MA* membrane area, *HRT* hydraulic retention time, *MLSS* mixed liquor suspended solids, *UF* ultrafiltration, *SRT* sludge retention time

Biological treatment has been identified as critical to the degree of micropollutants removal (Falås et al. 2012). Although, bioremediation processes are the most attractive and successful clean-up technologies, they are characterized by some disadvantages. Among them: (1) potential disruption of bioremediation additives by indigenous microflora, (2) poor susceptibility of some xenobiotic to biodegradation, (3) almost impossible removal of genetically modified microorganisms (GMMs) after bioremediation periods, (4) formation of intermediates characterized by high or indefinite toxicity or persistence, (5) difficulties with extrapolating the lab-scale experiments to field operations, and (6) occurring of contaminations as solids, gases, or liquids, which frequently impede the bioremediation processes should be mentioned. What is equally important is that microorganisms with increased degradation abilities often require suitable environmental growth conditions as optimal temperature, pH value, the presence of additional source of carbon, and appropriate levels of xenobiotics to induce appropriate enzymes. Additionally, such microorganisms frequently have to be adapted to increasing concentrations of contaminations, which may extend the bioremediation procedure (Kumar et al. 2011; Singh and Kumar 2016).

Up to now, several microorganisms able to use paracetamol and ibuprofen as a sole carbon and energy source have been described; however, metabolic pathways of biodegradation, especially for ibuprofen, remain poorly characterized. Two key metabolites formed during microbial degradation of paracetamol, hydrolytic phenolic dead-end metabolite 4-aminophenol and hydroquinone, have been identified (Hu et al. 2013; Zhang et al. 2013). It is also known that microbial degradation of ibuprofen may proceeds via ligation with coenzyme A and formation of isobutylcatechol or by direct trihydroxylation of the aromatic ring, which is prerequisite for further ring cleavage (Murdoch and Hay 2005; Murdoch and Hay 2013).

The main aim of the study was to summarize the newest knowledge about occurrence, toxicity, degradation pathways, and genes encoding enzymes involved in biotransformation/biodegradation of paracetamol and ibuprofen as the most emerging contaminants. To complete the overall picture of degradation processes, it is necessary to perform deep genetic and molecular analysis, which enables the isolation of mutants with increased degradation capabilities. The review presents robust analysis of paracetamol and ibuprofen prevalence in the environment and their toxicity underling the great importance of swift and consequent actions to decrease the presence of these drugs in the environment mainly by reduction of their release and efficient biodegradation. Bacterial strains and conditions used in experiments regarding biodegradation of paracetamol and ibuprofen as well as the genetic aspect of biodegradation possesses have been described.

## Sources of pharmaceuticals in the environment

Pharmaceuticals occurred in the environment mainly through the domestic wastewater, hospital effluents, industrial wastewater from pharmaceuticals production, run-off from aquacultures and concentrated animals feeding operations, and fish farming as well as a rural run-off and manure (Luo et al. 2014; Chiarello et al. 2016; Wiest et al. 2018). In recent years, intensified attention has been paid to the evaluation of consumer disposal patterns. Studies based on the questions survey showed that significant percentage of consumers store a large volume of unused pharmaceuticals and usually dispose of them through municipal trash. Researchers also highlighted that climatic conditions regulate pharmaceuticals input in certain periods as well as administration of a particular drug in common local diseases. On the other hand, as Vatovec et al. (2017) suggested the increase of social awareness about pro-environment behaviors, such as purchasing over-the-counter medication in smaller quantities and utilizing drug take-back programs could significantly minimize the growing pollution of environment. The release of sewage effluents was identified as a main route of surface waters contamination, since the wide range of pharmaceuticals and personal care products (PPCPs) are ubiquitously detected in treated wastewater (Luo et al. 2014; Arpin-Pont et al. 2016). Micropollutant contamination of groundwater is caused mainly by landfill leachate ground and surface water interaction, artificial recharge using reclaimed water, and infiltration of contaminated water from agricultural land. Pharmaceuticals present in solid waste discharge, sewage sludge, or manure fertilizer may leach from the fields and due to their mobility in the soil end up in groundwater (Arpin-Pont et al. 2016). Determination of drugs concentration in drinking water is difficult mainly due to the limits in quantification (LOQ) and limits in detection (LOD). Therefore, to study pharmaceutical occurrence in drinking water, advanced analytical techniques should be applied (Wiest et al. 2018). To date, for most of organic micropollutants, including paracetamol and ibuprofen, there are no discharges guidelines and monitoring recommendations. Moreover, synergistic, additive, and antagonistic effects between pharmaceuticals present in the environment are currently completely ignored (Luo et al. 2014). Since paracetamol and ibuprofen are one of the most popular over-the-counter drugs, co-occurrence of these pharmaceuticals in hospital or municipal wastewater as well as surface waters have been reported. As an example, the Yamuna River, which is the major tributary of Ganges River, can be given (Mutiyar et al. 2018). Kosma et al. (2010) reported the presence of 11 pharmaceuticals including paracetamol and ibuprofen in the municipal and hospital wastewater treatment plants of Ioannina City, located in Western Greece. Ashfaq et al. (2017a) investigated the occurrence of 11 frequently formulated drugs in the different environmental matrices near pharmaceutical formulation units of Shiekhupura, Lahore, Pakistan. Among them, paracetamol and ibuprofen were characterized by almost the highest detected concentrations. Chinnaiyan et al. (2018) placed paracetamol and ibuprofen on the list of the compounds relevant for India as emerging contaminants detected in water of a developing country examined by considering the data pertaining to pharmaceutical consumption data.

## Monocyclic nonsteroidal anti-inflammatory drugs

### Paracetamol

#### Occurrence and toxicity

Paracetamol (abbreviated as APAP, IUPAC name *N*-(4-hydroxyphenyl)ethanamide, acetaminophen, 4-acetaminophenol, 4′-hydroxyacetanilide, *N*-acetyl-*p*-aminophenol), is currently one of the most widely used over-the-counter available antiphlogistic drug worldwide (Wu et al. 2012; Rhee et al. 2013). Chemically, paracetamol consists of a benzene ring core substituted by one hydroxyl group and the nitrogen atom of an amide group at the (1,4) *para* position (Bales et al. 1985; Wu et al. 2012). In the UK, paracetamol is classified in the top three of the most frequently prescribed drugs. In the USA, where paracetamol is one of the 200 most frequently used pharmaceuticals its consumption during 2001–2005 reached about 29 billion doses (Li et al. 2014; Marchlewicz et al. 2015). Prevalent occurrence of paracetamol, as well as its main degradation product (4-aminophenol) in the environment, is also partly associated with their use for manufacturing of azo dyes and photographic materials (Zhang et al. 2013). Chong et al. (2017) evaluated consumers’ usage patterns of acetaminophen and according to the results, majority of consumers use APAP for a headache, fever, and common pain. Despite the fact that paracetamol is considered as generally safe, it is one of the most common drugs responsible for poisoning and liver damage. After administration, the majority of paracetamol is rapidly transformed in the liver by the conjugating enzymes urine 5′-diphosphoglucuronosyltransferase and sulfotransferases (SULTI A1 and SULTI A3) to the non-toxic compounds, followed by renal and biliary excretion. The remaining paracetamol is oxidized by phase I cytochrome P450 isoenzymes to the highly reactive and electrophilic metabolite, *N*-acetyl-*p*-benzoquinone-imine (NAPQI). NAPQI is detoxified by glutathione (GSH) to form cysteine- and mercapturate-paracetamol conjugates. With paracetamol overdose, GSH reserves are depleted, and as a consequence, NAPQI is accumulated, which results in covalent modification of thiol groups of proteins, nucleic acids damage, oxidation of membrane lipids, cell necrosis, and death (Pu et al. 2016; Van Wijk et al. 2017).

The increasing concentrations of paracetamol and other emerging contaminants result in the possibility of occurrence of toxic phenomena in non-target species present in receiving aquatic environments. Recently, the Environment Agency (EA) of England and Wales, due to the potential risk of pollutants to the aquatic environment, proposed ranking system with the list of the top 10 compounds, where paracetamol have been classified in 5th place (Ebele et al. 2017). This classification illustrates general change of approach in the legislation, e.g., diclofenac, another NSAID with polycyclic aromatic structure, has been included in the *Watch List*, which contains the most important candidates for a supplemented list of priority substances for the WFD (European Water Framework Directive, 2013). Nunes et al. (2015) highlighted that due to the extremely high dispersion of paracetamol in nature its environmental fate and ecotoxicological assessment should be a priority.

Paracetamol has been detected in different environmental matrices, e.g., surface water at concentrations as high as above 65 μg/L in the Tyne River, England (Roberts and Thomas 2006), higher than 78 μg/L in the Danube River (Grujíć et al. 2009), in an average concentration of 5 ng/L in different surface water (Esterhuizen-Londt et al. 2016), 12–64 μg/L and 1.57–56.9 μg/L in wastewater from Korea, Spain, and Western Balkan Region (Bosnia and Herzegovina, Croatia, and Serbia), up to 483 μg/kg in sludge and up to 81 μg/kg in soil (Luo et al. 2014; Ashfaq et al. 2017a, b). Moreover, paracetamol easily accumulates in aquatic environment and it is known to exhibit virtually no sorption nor retardation in aquifer sand studies (De Gusseme et al. 2011). Even though the detected concentrations of paracetamol and other NSAIDs range from nanograms per liter in sewage effluents to micrograms per liter in natural waters, a variety of potential negative effects for these low levels have been described including, e.g., reproductive or DNA damage, accumulation in tissues, oxidative stress, lipid peroxidation, and behavioral changes observed in algae, microcrustaceans, mollusks, or teleost fish (Islas-Flores et al. 2013; Gómez-Oliván et al. 2014; Minguez et al. 2016; Islas-Flores et al. 2017) (Table 2). Oliveira et al. (2015) and Ramos et al. (2014) highlighted that the available data about the chronic effects and action of pharmaceuticals, which are calculated for environmentally relevant concentrations showed that most of pharmaceuticals at these levels are not responsible for lethality. Thus, evaluation of the toxicity based on sub-lethal endpoint tests will be far better than using organisms from different trophic levels. Currently, the most commonly tested biomarkers, which are used as early indicators for even very low concentration of pollutants, are enzymatic markers of different metabolic pathways, e.g., oxidative stress or neuronal function.

**Table 2:** Toxicity of paracetamol and ibuprofen to different aquatic organisms

Organism	Compound	Tested concentrations	Exposure time	Toxicity	LC/EC/IC	ISO/OECD	Reference
*Daphnia magna*	Paracetamol	0.00001, 0.001, 0.25 mg/L	48 h	Significant inhibition of acetylcholinesterase and selenium-dependent glutathione peroxidase enzymes	–	ISO 6341:1996	Oliveira et al. (2015)
*Mytilus galloprovincialis*	Paracetamol	20, 200 μg/L	10 days	Significant increase of hepatic lipid peroxidation levels and inhibition of acetylcholinesterase activity in gill	–	–	Solé et al. (2010)
*Anguilla anguilla*	Paracetamol	5, 25, 125, 625, 3125 μg/L	96h	Inhibition of acetylcholinesterase activity, no response in catalase and lactate dehydrogenase enzymes activity	–	–	Nunes et al. (2015)
*Brachionus koreanus*	Paracetamol	0.1, 1, 10, 100, 1000, 10,000, 100,000 μg/L	24h	Inhibition of acetylcholinesterase activity	–	–	Rhee et al. (2013)
*Oncorhynchus mykiss*	Paracetamol	0.05, 0.5, 5 mg/L (acute exposure), 12.5, 25, 50 μg/L (chronic exposure)	96 h (acute exposure), 28 days (chronic exposure)	Increase in catalase and glutathione peroxidase activity	–	–	Ramos et al. (2014)
*Oreochromis mossambicus*	Paracetamol	500 mg/kg body weight	24 h	Increased glutamate oxaloacetic transaminase, glutamate pyruvic transaminase, alkaline phosphatase, acid phosphatase, glucose-6-phosphate dehydrogenase, lactate dehydrogenase, superoxide dismutase, catalase, glutathione peroxidase, glutathione reductase, glutathione *S*-transferase, lipid peroxidase and reduced glutathione enzymes activity	–	–	Kavitha et al. (2011)
*Cyprinus carpio*	Paracetamol	100 μg/L	96 h	Induction of oxidative stress, increase in glutathione peroxidase activity	LC_50_ > 1000 μg/L	–	Nava-Álvarez et al. (2014)
*Rhamdia quelen*	Paracetamol	0.25, 2.5 μg/L	21 days	Disruption of hypothalamic-pituitary-gonadal axis, reduction of hemoglobin and hematocrit, increase in leukocytes and thrombocytes, reduction of testosterone and increase in estradiol levels, induction of oxidative stress, and hepatotoxicity	–	–	Guiloski et al. (2017)
*Diopatra neapolitana*	Paracetamol	5, 25, 125, 625, 3125 μg/L	39 days	diminished regenerative capacity in a dose-dependent manner	–	–	Freitas et al. (2015)
*Daphnia magna*	Paracetamol	0.08 to 6.48 mg/L	72 h (acute toxicity test), 21 days (chronic exposure)	Significant concentration-dependent adverse effects on total number of broods per female	LC_50_ - 224 mg/L (24 h), 40 mg/L (48 h), 8.06 mg/L (72 h), 5.32 mg/L (21 days), EC_50_ - 4.78 mg/L (body length), 4.21 mg/L (carapaces per adult), 2.38 mg/L (broods per female), 1.12 mg/L (egg production per female)	OECD TG 202 (1984); OECD TG 211 (1998)	Du et al. (2016)
*Dreissena polymorpha*	Ibuprofen	0.2, 2.0, 8.0 μg/L	96 h	Induction of moderate genetic and cellular damage, imbalance in the activity of catalase, superoxide dismutase, glutathione peroxidase and glutathione *S*-transferase	14-day EC_50_ for reproduction rate - 13.4 mg/L	–	Parolini et al. (2011)
*Dreissena polymorpha*	Ibuprofen	250 ng/L	2 weeks	induction of Significant transitory antioxidant defense responses and membrane damage in the digestive gland	–	–	Gonzales-Rey and Bebianno (2012)
*Oryzias latipes*, *Daphnia magna*, *Moina macrocopa*, H295R cell line	Ibuprofen	0.02, 0.2, 2, 20 mg/L (H295R cells); 3.13, 6.25, 12.5, 25.0, 50.0 mg/L (*D. magna*, *M. macrocopa*); 0.01, 0.1, 1, 10, 100, or 1000 g/L (*O. latipes*)	24h and 48h (H295R cells), 21 days (*D. magna*), 7–8 days (*M. macrocopa*), 144 days (*O. latipes*)	Increased 17β-estradiol production and aromatase activity in H295R cells, delay in hatching of eggs in *O. latipes* and induction of vitellogenin in males, negative effects on reproduction in *D. magna* and *M. macrocopa*	*D. magna*, 48 h immobilization EC_50_ - 51.4 mg/L, 21 days reproduction NOEC < 1.23 mg/L; *M. macrocopa*, 48 h immobilization EC_50_ - 72.6 mg/L, 7 days reproduction NOEC - 25 mg/L; *O. latipes*, 120 days survival NOEC - 0.0001 mg/L	*D. magna*: OECD TG 211 (2008); *O. latipes*: OECD TG 210 and 203 (1992)	Han et al. (2010)
*Gammarus pulex*	Ibuprofen	1 ng/L to 1 mg/L	1.5h	Decreased activity at low concentrations (1–100 ng/L), activity at higher concentrations (1 μg/L–1 mg/L) similar to the control	–	–	De Lange et al. (2006)
*Daphnia magna*	Ibuprofen	0.5, 5, 50 μg/L	6 and 48 h (enzymes activity and genes expression level measurements), 21 days (chronic exposure)	Significantly decrease in total amount of eggs, total number of brood per female, body length, increased activities of glutathione *S*-transferase, superoxide dismutase and catalase, inhibition at low concentration (0.5 μ/L) and induction at high concentration (50 μg/L) of CYP360A gene expression level, inhibition after short time of exposure (6 h) and induction with prolonged exposure time of CYP314 at low concentration (0.5 μg/L)	LC_50_ at 48 h - > 100 mg/L	OECD TG 202 (2004)	Wang et al. (2016)
*Cirrhinus mrigala*		30, 60, 90, 120, 150 mg/L	24 h (static acute toxicity measurement), 35 days (sub-lethal toxicity measurement)	Increased levels of hemoglobin, hematocrit, mean cellular volume, mean cellular hemoglobin, leukocytes, plasma glucose and alanine transaminase, mixed trend in aspartate aminotransaminase enzyme activity, (LC_50_ 142 mg/L, after 24 h)	LC_50_ for 24 h - 142 mg/L	–	Saravanan et al. (2012)
*Ruditapes philippinarum*	Ibuprofen	0.1, 5, 10, 50 μg/L	35 days	Diminished health status, alterations of blood parameters and hemocytes	EC_50_ of lysosomal membrane stability - 0.71 μg/L	–	Aguirre-Martínez et al. (2013)
*Asterias rubens*, *Psammechinus miliaris*, *Arenicola marina*	Ibuprofen	0.01 μg/L to 1 mg/L	Up to 1 h (*A. rubens* and *P. miliaris*), up to 2 h (*A. marina*)	Significant reduction of fertilization success of *P. miliaris* (> 1 mg/L), no effects in oocytes of *A. marina* and *A. rubens*	EC_50_ for *P. miliaris* gametes - 792.96 mg/L; EC_50_ for *A. marina* gametes > 10,000 mg/L	–	Zanuri et al. (2017)
*Allivibrio fischeri*	Ibuprofen	Serial dilution of the tested compound with dilution factor of 2	15 min	–	IC_50_ after 15 min of exposure - 18.3 mg/L	–	Di Nica et al. (2017)
*Navicula* sp.	Ibuprofen	0.1–100 mg/L	10 days	Growth stimulation, increased chlorophyll and carotenoids content at low concentrations of IBU (0.1–1 mg/L), decreased chlorophyll and carotenoids content at higher concentrations of IBU (10–100 mg/L)	–	OECD TG 201 (2011)	Ding et al. (2017)
*Scenedesmus rubescens*	ibuprofen	62.5, 250, 1000 μg/L	30 days	Growth inhibition, significant morphological and ultrastructural alterations, mainly large cytoplasmic inclusions, decrease of chlorophyll content and increase of carotenoids	–	–	Moro et al. (2014)
*Chlorella vulgaris*	Ibuprofen	35–320 mg/L	96 h	Significant effect on population density	IC_50_ - 89.65 mg/L	OECD TG 201 (2006)	Geiger et al. (2016)
*Acutodesmus obliquus, Chlamydomonas reinhardtii*, *Nannochloropsis limnetica*	Ibuprofen	0.003, 0.03, 5, 100, 500, 1000 mg/L	5 h	Sensitivity of algae dependent on the cellular phosphorus status	*A. obliquus* EC_50_ - 288 mg/L, *N. limnetica* EC_50_ - 965 mg/L, *C. reinhardtii* EC_50_ - 622 mg/L	–	Grzesiuk et al. (2016)
*Daphnia magna*	Ibuprofen	0.4 to 32.4 mg/L	72 h (acute toxicity test), 21 days (chronic exposure)	Significant concentration-dependent adverse effects on total number of broods per female	LC_50_ - 116 mg/L (24 h), 23.5 mg/L (48 h), 8.33 mg/L (72 h), 3.97 mg/L (21 days), EC_50_ - 2.51 mg/L (body length), 1.77 mg/L (carapaces per adult), 1.63 mg/L (broods per female), 0.7 mg/L (egg production per female)	OECD TG 202 (1984); OECD TG 211 (1998)	Du et al. (2016)

*LC* lethal concentration, *EC* effective concentration, *IC* inhibitory concentration, *OECD* Organization for Economic Co-operation and Development, *ISO* International Organization of Standardization

The mechanism of paracetamol toxicity seems to be highly conserved and it is believed to act in the same way in the vertebrates as in the invertebrates. First, an extremely important parameter from the ecological point of view concerns its neurotoxicity. Oliveira et al. (2015) analyzed the effects of paracetamol on biomarkers of neuronal regulation and enzymatic oxidative stress defense, including total cholinesterases (ChEs), catalase (CAT), glutathione *S*-transferases (GSTs), and total and selenium-dependent glutathione peroxidase activities (total GPx; Se-GPx), respectively. As the obtained results indicated, after exposure to APAP at concentration of 10 μg/L significant inhibition of acetylcholinesterase (AChE) and Se-GPx activity in *Daphnia magna* was observed. Similar results were obtained by Solé et al. (2010) for seawater mussel *Mytilus galloprovincialis* after exposure to paracetamol at concentration ranging from 0.023 to 0.403 mg/L. It was a first report of anticholinesterasic effect caused by paracetamol. Oliveira et al. (2015) noticed that some authors postulate that enzyme inactivation may be the result of oxidative stress, which is the most common effect of paracetamol exposure (Nunes et al. 2015; Ramos et al. 2014). The link between the oxidation and cholinesterase activity was shown by Weiner et al. (1994), by proving that oxidative conditions altered the conformation of acetylocholinesterase of the fish *Torpedo californica*, and Delwing-de Lima et al. (2010), who showed that neuronal enzymatic biomarkers from rodents could be oxidized in vivo. Neurotoxic effect of paracetamol, namely inhibition of AChE activity, was also confirmed by Rhee et al. (2013) for rotifer species *Brachionus koreanus*. These results illustrate quite unexpected neurotoxic effects of well-known oxidant pharmaceutical pollutions, including paracetamol. Moreover, Nunes et al. (2015) showed that even a moderate oxidative stress caused by paracetamol may result in neurotransmission impairment in the European eel *Anguilla anguilla*.

Many authors showed that paracetamol causes the significant increase in enzymatic biomarkers involved in cellular redox system, which confirmed that paracetamol triggers oxidative changes. Measurement of glutathione peroxidase activity (GPx) serves as a biomarker of oxidative stress due to the role of glutathione enzymes family in removal of some reactive oxygen species (ROS; mainly hydrogen peroxide and superoxide radicals). The second biochemical assay most commonly used for evaluation of oxidative stress is measurement of catalase activity. However, in studies concerning the oxidative stress caused by paracetamol a lot of contradictory results have been published. Oliveira et al. (2015) and Monteiro et al. (2006) suggested that the decrease in GPx activity after exposure to paracetamol is related to the high amount of the hydroperoxide products formed during lipid peroxidation, which exceeds the antioxidant capacity of GPx. Secondly, the decrease in GPx activity may be caused by direct inactivation of enzyme by reactive metabolites of APAP. The increase in GPx activity in response to paracetamol exposure has been documented for *Orcheochromis mossambicus* (Kavitha et al. 2011), *Cyprinus carpio* (Nava-Álvarez et al. 2014), or *Dreissena polymorpha*, where the increase of other antioxidant enzyme activity, mainly catalase and superoxide dismutase, has been observed (Parolini and Binelli 2012). Nunes et al. (2015) evaluated a battery of biochemical effects occurring after exposure to paracetamol in the European eel *Anguilla anguilla*. As shown in the obtained results, paracetamol was responsible for the increase in GSTs activity in the liver, while the CAT activity was not altered. This suggests that GSTs activity may be related to detoxification mechanisms, namely conjugation with GSH, which is mainly responsible for paracetamol excretion. On the other hand, in the gills, GSTs activity was significantly decreased. Therefore, the authors suggested that in the combination with the increase in lipid peroxidation, in the same tissue, the occurrence of mild oxidative stress cannot be excluded. No changes in the activity of lactate dehydrogenase (LDH) were reported, which confirmed that paracetamol exposure is not related to anaerobic metabolism. Ramos et al. (2014) evaluated the oxidative stress caused by paracetamol in a model standard organism rainbow trout, *Oncorhychus mykiss*. Tested concentrations of APAP in acute exposure (96 h) were sub-lethal: 0.00, 0.05, 0.50, and 5.00 mg/L. To test chronic exposure (28 days), tested concentrations of APAP were 0.00, 12.5, 25.0, and 50.0 μg/L. As shown in the obtained results, catalase activity as well as selenium-dependent and total GPx were increased in both tested periods. Glutathione reductase (GRed) activity was more strongly expressed after chronic exposure, whereas metabolism of GSH was affected after both periods of exposures. In a dose-dependent manner, a significant increase in lipid peroxidation was also shown. It is extremely important to notice that oxidative changes and peroxidative damage were observed at realistic environmental concentrations reported for some freshwater compartments. As was mentioned above, GRed activity responsible for the maintenance of cytosolic physiological concentrations of GSH was increased, which strongly confirmed that most of the antioxidant defense is located in the liver.

Ashfaq et al. (2017a, b) evaluated the ecological risk assessment expressed as the ratio between the predicted environmental concentration (PEC) and measured environmental concentration (MEC) to the predicted no-effect concentrations (PNEC) of the pharmaceuticals, which generally approximates the harmful dose of various pharmaceuticals to different species living in the aquatic environments. This ratio is considered in terms of risk quotient (RQ) and hazard quotient (HQ). MEC is the maximum measured environmental concentration in μg/L and PNEC is the predicted no-effect concentration (μg/L). The value of RQ > 1 indicates high risk to the aquatic community whereas RQ < 1 indicates medium risk (Bouissou-Schurtz et al. 2014). For paracetamol, RQ values were about 64, 9.2, 5.0, and 0.11 against daphnia, *Streptocephalus proboscideus*, *D. magna*, and green algae, respectively. These results warrant high risk of paracetamol against daphnia, *S. proboscideus*, and *D. magna* and the medium risk against green algae.

Guiloski et al. (2017) demonstrated the disruption of hypothalamic-pituitary-gonadal axis and severe changes of hematological parameters, i.e., mild blood congestion, leukocyte infiltration, and reduction of hemoglobin and hematocrit in male fish of *Rhamdia quelen* after exposure to environmental concentrations of paracetamol in a semi-static bioassay during 21 days. Freitas et al. (2015) investigated the effects of ecologically relevant concentrations (25 μg/L) of paracetamol on Polychaete *Diopatra neapolitana* regenerative tissue capacity. The obtained results revealed that paracetamol significantly decrease the regenerative capacity in a dose-dependent manner. Du et al. (2016) showed significant time-dependent and concentration-dependent adverse effects of paracetamol with 58.3% mortality of *D. magna*. Moreover, paracetamol caused 50% mortality of *D. magna* after 21 days of exposure at the concentration of 5.32 ± 0.32 mg/L. According to the previous EU Directive 93/67/EEC (Commission of the European Communities, 1996), which classified chemicals on the basis of the EC_50_ values, paracetamol was “harmful to the aquatic organisms” (EC_50_ concentration between 11 and 100 mg/L) and “very toxic to the aquatic organisms” (EC_50_ concentration < 1 mg/L) after long-term exposure. The currently applicable legal act regulating the responsibility from public authorities to industry with regard to assessing and managing the risks posed by chemicals and providing appropriate safety information for their users is 1907/2006 the European Regulation on Registration, Evaluation, Authorisation and Restriction of Chemicals - REACH (Regulation (EC) No 1907/2006).

#### Biotransformation/biodegradation of paracetamol

Currently, the wastewaters which contain pharmaceuticals are mainly treated with the advanced oxidation processes (AOP) including Fenton and photo-Fenton processes, photocatalysis with titanium dioxide, UV photolysis, or ozonation coupled with H_2_O_2_. The reported efficiency of paracetamol removal in WWTPs is estimated from almost complete to as high as 86% for municipal sewage (De Gusseme et al. 2011). Nevertheless, knowledge about the further fate of paracetamol in the environment is still limited. Ahmed et al. (2017) and Glanclément et al. (2017) summarized the chemical and the biological treatment technologies and reported that a hybrid system based on ozonation followed by treatment with biological activated carbon was found to be the most efficient in the removal of pesticides, beta-blockers, and pharmaceuticals. However, despite the high efficiency of chemical treatment methods, the high operational costs, harsh reaction conditions, and formation of secondary metabolites with high or indefinite toxicity associated with these methods often make them not a desirable choice. Therefore, biodegradation and/or biotransformation of pharmaceuticals, including NSAIDs, with the use of bacterial strains with enhanced degradation abilities is a promising environmentally and economically sustainable tool in wastewater treatment. The capability of microorganisms to degrade xenobiotics depends on various environmental factors, affecting degradation processes. It is widely known that temperature plays an important role in xenobiotics degradation by affecting bacterial physiology and velocity of enzymatic reaction. The maximal biodegradation rate of xenobiotics occurs at the temperature of 30–40 °C. At lower temperatures, bacterial membranes become more rigid, as the result in increased viscosity of membrane phospholipids. On the other hand, at higher temperatures, the membrane transport is frequently hindered as the result of membrane-associated proteins denaturation. The second significant factor, which regulates the degradation of xenobiotics is pH, since it affects microbial cell morphology, activity, and membrane properties. Through the changes in ionization of pharmaceuticals molecules, pH may also influence biosorption and toxicity of pollutants. For paracetamol, at lower pH, the formation of protonated form (ROH) has been observed, whereas in alkaline environment paracetamol occurred as phenolate (RO^−^) form. Since pK_a_ of paracetamol is 9.5, at slightly alkaline environment, APAP is present in the non-ionic form (Xagoraraki et al. 2008). These allow supposition that the highest degradation rate of paracetamol may occur in neutral pH value. This supposition was confirmed for *Pseudomonas moorei* KB4 strain, for which the optimal pH for paracetamol degradation was 7.0 (Żur et al. 2018).

The increased ability to degrade organic micropollutants is related also with the structure of bacteria cell membranes, i.e., the presence of lipopolysaccharide (LPS) and outer membrane in Gram-negative bacteria as well as specific cell wall architecture in Gram-positive bacteria. Moreover, bacteria are able to form biofilms embedded with extracellular polymeric substances (EPS), increasing their protection against harsh environmental conditions, and surface active agents (biosurfactants) increasing their access to hydrophobic impurities, or specific enzymes, which are responsible for xenobiotics degradation. The complexity of polluted environment in a natural way influences the ability of indigenous and introduced microorganisms to xenobiotics degradation. The conditions of a particular environment must enable microbial growth and activity. As the bioremediation is a part of whole cell metabolism and results from the activity of complex microflora present in the polluted area, it is generally known that the co-occurrence of wide range of organic pollutants (e.g., PPCPs, lipid regulators, alkylating agents, hormones) and introduction of an additional carbon source to the culture may enhance metabolism of xenobiotics (Kumar et al. 2011; Singh and Kumar 2016). Micropollutants related factors (e.g., bioavailability, volatility, hydrophobicity, acidity determined by functional groups, chemisorption, electrostatic adsorption, charge repulsion, the presence of functional groups donating electrons) in a significant way regulates biodegradation processes. Up to now, only a few bacterial strains able to degrade paracetamol have been isolated (Table 3). Ahmed et al. (2001) described *Pseudomonas* sp. ST1 strain isolated from contaminated sites in Bhai Pheru, Pakistan, being able to grow on paracetamol and its main degradation intermediate, 4-aminophenol, at concentration of 4 g/L as a sole carbon and energy source and 5 g/L in medium supplemented with glucose. In the degradation experiments, ST1 strain reduced 777 mg/L of paracetamol during 72 h and 651 mg/L of 4-aminophenol during 48 h. 4-aminophenol, classified as dead-end metabolite is characterized by significant nephrotoxicity, mutagenic and teratogenic effects, and ability to induce DNA cleavage in mouse and human lymphoma cells. Similar to acetaminophen, its first hydrolytic product is poorly biodegradable and may inhibit degradation metabolic pathway and indirectly slow down or completely stop the bioremediation processes. Moreover, for paracetamol, co-contaminants occurring in the polluted environments, i.e., 4-hydroxybenzoate or 4-chlorophenol may compete with 4-aminophenol formed during degradation of paracetamol, since the same enzymes are engaged in degradation of *para*-substituents (Guzik et al. 2013b). Up to now, only several microorganisms able to degrade 4-aminophenol have been described. Several authors reported that 4-aminophenol may be a key metabolite in the microbial degradation of nitrobenzenes and amines (Takenaka et al. 2003). Khan et al. (2006) isolated *Pseudomonas* sp. ST-4 strain able to grow in the presence of 4-aminofenol in the co-metabolic culture with glucose at concentrations reaching 400 mg/L. The induced by 4-aminophenol cells were later able to reduce 80% (50 mg/L) of the initial concentration of the tested compound.

**Table 3:** Degradation of paracetamol, 4-aminophenol, and ibuprofen by bacteria and their consortia

Strain/microbial consortium	Compound	Degraded concentrations/time of degradation	Metabolites	Time of experiment	Conditions of degradation	Other information	Reference
*Pseudomonas* sp. ST1	Paracetamol	777,000 μg/L	nd	72 h	Exponential phase cells grown on the tested compounds were harvested and resuspended in fresh buffer. Degradation was carried out in flasks incubated at 30 °C in a shaking incubator at 150 rpm	Strain was isolated by enrichment culture. Paracetamol was used as the sole carbon and energy source	Ahmed et al. (2001)
*Pseudomonas* sp. ST1	4-Aminophenol	651,000 μg/L	nd	48 h	Exponential phase cells grown on the tested compounds were harvested and resuspended in fresh buffer. Degradation was carried out in flasks incubated at 30 in a shaking incubator at 150 rpm	Strain was isolated by enrichment culture. 4-Aminophenol was used as the sole carbon and energy source	Ahmed et al. (2001)
*Pseudomonas* sp. ST-4	4-Aminophenol	40,000 μg/L	nd	72 h	Degradation was performed in flasks with mineral salt medium (PNR-G) with required 4-aminophenol concentrations incubated at 30 °C with shaking at 100 rpm	Strain was isolated by enrichment culture. Able to grow on 4-aminophenol at concentration up to 400 mg/L on mineral salt media plates. For enhanced metabolic properties of strain before degradation experiments 4-aminophenol at a concentration of 50 mg/L was used as an inducer	Khan et al. (2006)
*Burkholderia* sp. AK-5	4-Aminophenol	11 ± 0.2 mM	1,2,4-Tri-hydroxyl-benzene, 1,4-benzenediol, maleylacetic acid	16 h	Strain was grown in mineral basal medium with 4-aminophenol at concentration of 1.2 g/L in a 500 mL flasks with shaking at 30 °C	Strain was isolated from the rice filed soil by enrichment culture and utilizes 4-aminophenol as the sole carbon and energy source	Takenaka et al. (2003)
*Delftia tsuruhatensis*	Paracetamol	From 10.325 ± 0.027 to 0.263 ± 0.034 mg/L	Hydroquinone	48 h	Degradation was performed in minimal medium with 1% of inoculum (*v*/*v*)	Isolated from the membrane bioreactor (MBR) with a working volume of 20 L and 3 membrane plates by enrichment culture	De Gusseme et al. (2011)
*Pseudomonas aeruginosa*	Paracetamol	From 6.152–to 0.083 mg/L during	Hydroquinone	48 h	Degradation was performed in minimal medium with 1% of inoculum (*v*/*v*)	Isolated from the membrane bioreactor (MBR) with a working volume of 20 L and 3 membrane plates by enrichment culture	De Gusseme et al. (2011)
*Rhodococcus ruber* IEGM77	Paracetamol	Almost 100% for pills in K medium, 100% for pills in RS medium, almost 30% for pure substance in K medium, 20% for pure substance in RS medium	4-Aminophenol, hydroquinone, pyrocatechol	20 days for pills in K medium, 10 days for pills in RS medium, 20 days for pure substances in both media	Biodegradation experiment was carried out under batch conditions in 250 mL flasks, with shaking at 150 rpm and 28 °C. Paracetamol was added as commercial pills (0.2 g of paracetamol and 10% of different adjuvants) and as the pure substance	For induction of oxygenases strain was plated on agar mineral medium with *n*-hexadecane. For degradations studies APAP was used as the sole carbon and energy source	Ivshina et al. (2006)
*Pseudomonas moorei* KB4	Paracetamol	50 mg/L of paracetamol	4-Aminophenol, hydroquinone, 4-hydroxymuconic semi-aldehyde	24 h	Degradation experiments were performed in mineral salts medium supplemented with glucose (0.1%) at 30 °C with shaking at 130 rpm	Strain KB4 is also capable to degrade 4-aminophenol	Żur et al. (2018)
Microbial consortium from MBR	Paracetamol	85% of an average 20μg/L/day during the period A (days 1–15 with HRTs 3) > 99% of 105.7 μg/L/day during the period E (days 61–75 with HRTs 1)	nd	15 days, 14 days	NEC inoculated with the MBR system with paracetamol as the sole carbon and energy source. 60 days (period A–D) with a HRTs 5 days was applied as an adaptation phase	During all periods, ammonium and some nitrite were still detected in the effluent of the bioreactor. Decreasing ammonium concentration from 19.8 mg NH_4_^+^-N/L in period A to 1.2 mg NH_4_^+^-N/L in period D and E showed no influence on the APAP removal efficiency	De Gusseme et al. (2011)
Biomass from MBR in batch incubation experiments	Paracetamol	More than 99% from initial 1097.6 ± 40.0 μg/L	nd	72 h	The biomass from MBR was harvested and incubated in mineral salt medium with paracetamol as the sole carbon and energy source and NH_4_^+^-N/L at concentration of 52.5 mg. After the lag phase which lasted 6 h, 64% removal of paracetamol was observed	No degradation was detected in the abiotic control and in the experiment with heat-inactivated biomass from MBR	De Gusseme et al. (2011)
*Pseudomonas aeruginosa* HJ1012	Paracetamol	2200 mg/L	4-Aminophenol, hydroquinone, formic acid, oxalic acid, lactic acid, succinic acid, nitrate nitrite	75 h	Batch experiments were performed in mineral medium with required paracetamol concentrations incubated in the dark at 30 °C with shaking at 160 rpm	Strain was isolated from the microbial aggregate from the sequencing batch reactor (SBR) able to paracetamol degrade. Paracetamol used as the sole carbon and energy source	Hu et al. (2013)
*Stenotrophomonas* sp. f1	Paracetamol	400 mg/L	4-Aminophenol, hydroquinone, formic acid, oxalic acid, lactic acid, succinic acid, nitrate, nitrite	116 h	Batch experiments performed in mineral salts medium supplemented with paracetamol with required concentrations	Strain isolated from paracetamol-degrading aerobic aggregate. Paracetamol used as the sole carbon, nitrogen and energy source. The increased paracetamol concentration as high as 600 mg/L inhibited the f1 strain growth	Zhang et al. (2013)
*Pseudomonas* sp. f2	Paracetamol	2500 mg/L	4-Aminophenol, hydroquinone, formic acid, oxalic acid, lactic acid, succinic acid, nitrate, nitrite	70 h	Batch experiments performed in mineral salts medium supplemented with paracetamol with required concentrations	Strain isolated from paracetamol-degrading aerobic aggregate. Paracetamol used as the sole carbon, nitrogen and energy source. Paracetamol at concentration of 3000 mg/L completely stopped the degradation	Zhang et al. (2013)
*Pseudomonas* sp. fg-2	Paracetamol	2000 mg/L	4-Aminophenol, hydroquinone, formic acid, oxalic acid, lactic acid, succinic acid, nitrate, nitrite	45 h	Batch experiments performed in mineral salts medium supplemented with paracetamol with required concentrations	Strain isolated from paracetamol-degrading aerobic aggregate. Paracetamol used as the sole carbon, nitrogen and energy source	Zhang et al. (2013)
Consortium composed of f1, f2, and fg-2 strain	Paracetamol	4000 mg/L	4-aminophenol, hydroquinone, formic acid, oxalic acid, lactic acid, succinic acid, nitrate, nitrite	130 h	Batch experiments performed in mineral salts medium supplemented with paracetamol with required concentrations	Authors suggested occurring the synergistic interaction between strains in the consortium, which results in higher tolerance towards paracetamol than in the case of the individual strain	Zhang et al. (2013)
*Sphingomonas* sp. Ibu-2	Ibuprofen	500 mg/L of R/S enantiomers of ibuprofen mixture	Isobutyl-catechol, 5-formyl-2-hydroxy-7-methylocta-2,4-di-enoic acid, 2-hydroxy-5-isobutylhexa-2,4-dienedioic acid	80 h	Substrates specificity analysis (degradation) experiments were performed in mineral salts medium supplemented with 500 mg/L IBU and placed on a vertical rotor	Strain Ibu-2 is able to grow on ibuprofen used as the sole carbon and energy source	Murdoch and Hay (2005, 2013)
*Bacillus thuringiensis* B1(2015b)	Ibuprofen	Up to 20 mg/L in co-metabolic studies	2-hydroxy-ibuprofen, 2-(4-hydroxy-phenyl)propionic acid, 1,4-hydro-quinone, 2-hydroxy-1,4-quinol	6 days	Degradation experiments were performed in mineral salts medium supplemented with 1 mg/L of glucose for co-metabolic studies, at 30 °C with shaking at 130 rpm	Strain was isolated from the contaminated soil by enrichment culture. B1 strain is also able to use other aromatic compounds as the carbon and energy source, e.g., phenol, vanillic acid, protocatechuic acid, benzoic acid and 4-hydroxybenzoic acid	Marchlewicz et al. (2017a, 2017b) Marchlewicz et al. (2016)
*Patulibacter* sp. I11	Ibuprofen	125 μg/L 31 μg/L 46 μg/L	nd	300 h, 90 h, 90 h	Degradation performed in M9 medium supplemented with yeast extract and tryptone or in OD-2 medium supplemented with ibuprofen	Strain was isolated from wastewater treatment plant	Almeida et al. (2013)
*Variovorax* sp. Ibu-1	Ibuprofen	200 mg/L	Trihydroxyibuprofen	75 h	Degradation and growth analyses were performed in mineral salt medium supplemented with ibuprofen	Strain isolated from activated sludge from wastewater treatment plant using enrichment technique. Trihydroxy-ibuprofen probably is a dead-end metabolite	Murdoch and Hay (2015)
*Nocardia* sp. NRRL 5646	Ibuprofen	In biotransformation studies 1000 mg/L of each ibuprofen enantiomers were metabolized	Ibuprofenol, ibuprofenol acetate	120 h	nd	Strain NRRL 5646 is also able to reduce benzoic acid derivatives. The carboxylic acid reductase system was R-enantioselective	Chen and Rosazza (1994)

*nd* not determined

**Fig. 1:**
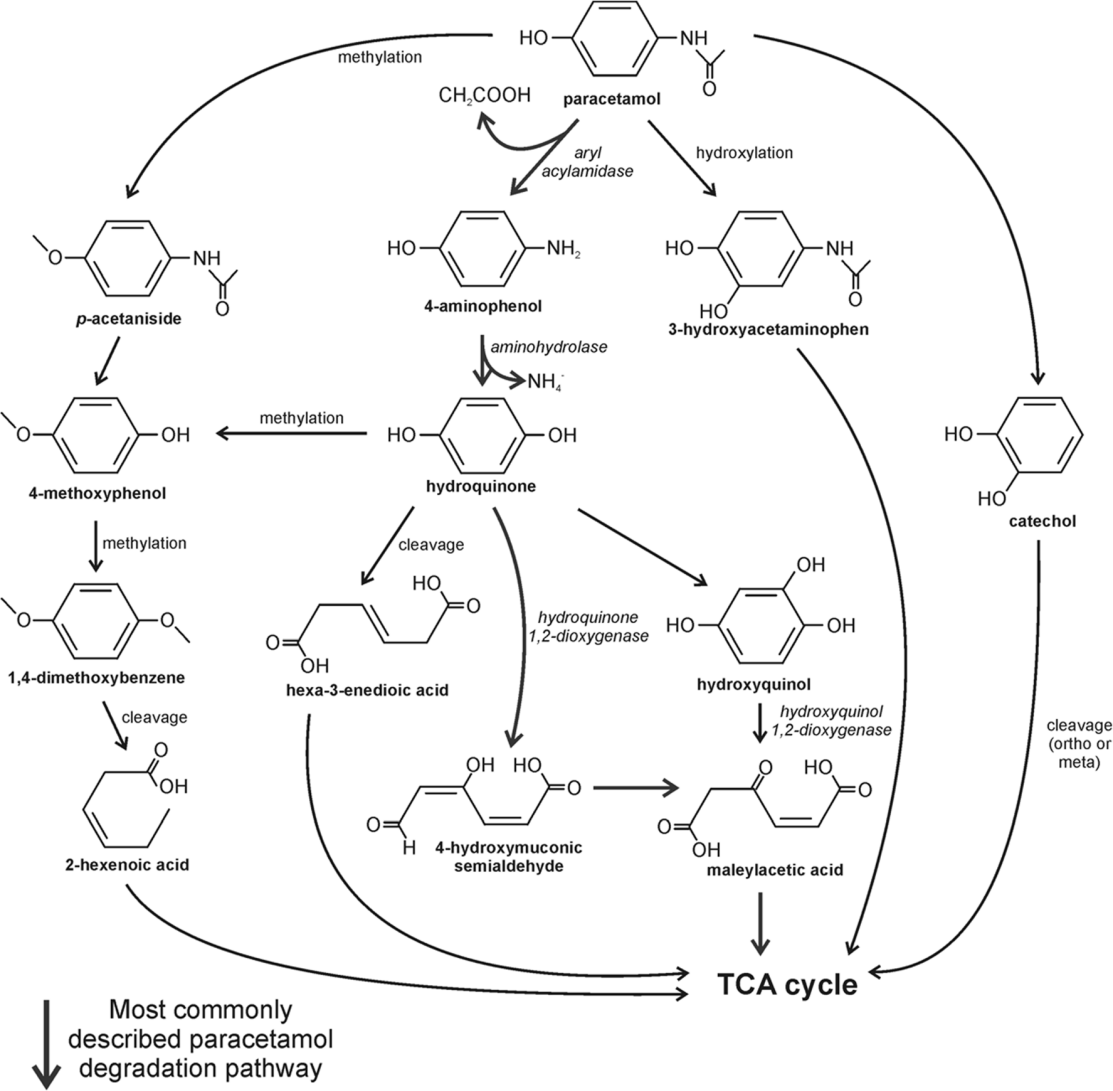
Biodegradation pathway of paracetamol (Hu et al. 2013; Zhang et al. 2013; Takenaka et al. 2003; Li et al. 2014)

De Gusseme et al. (2011) demonstrated the microbial removal of paracetamol in a membrane bioreactor (MBR), which was fed with paracetamol at concentration of 100 μg/L (average 20 μg/L of paracetamol per day) as the sole carbon and energy source for 16 days at a hydraulic retention time (HRT) of 5 days. After this period, more than 99.9% of the initial paracetamol concentration was degraded. Microbial consortium in the bioreactor was also shown to be able to remove paracetamol at environmentally relevant concentrations amounting 8.3 μg/L. Further analysis enable isolation of two paracetamol-degrading strains identified as *Delftia tsuruhatensis* and *Pseudomonas aeruginosa.* Microbiological degradation of paracetamol was performed using inoculum with an enriched nitrifying culture (NEC). The microbial biomass was able to remove paracetamol at concentration of 100 μg/L continuously at an hydraulic retention time (HRT) of 1 day. Moreover, batch incubation experiments with heat-inactivated bacteria confirmed that paracetamol removal was not the result of sorption to the biomass. Further experiments indicated that there was no influence of the matrix of a WWTP effluent on APAP degradation and that the biomass was capable of degrading APAP at environmentally relevant concentrations. Inhibited nitrification highlighted the significance of the associated heterotrophic bacteria. Moreover, during microbial degradation of paracetamol by two described isolates, a brown colorization of synthetic mineral medium was observed, which suggest the formation and/or accumulation of degradation intermediates, e.g., polymerization products of catechol. Further studies with *D. tsuruhatensis* strain showed that the main transformation product—hydroquinone—was also further metabolized, mainly due to the oxidative ring opening which results in loss of chromophoric structure. Hu et al. (2013) described strain HJ1012 classified as *Pseudomonas aeruginosa* which exhibit ability to degrade paracetamol at concentration up to 2.2 g/L and utilize it as the sole carbon and energy source. Eight intermediates including 4-aminophenol, hydroquinone, formic acid, lactic acid, oxalic acid, succinic acid, nitrate, and nitrite have been identified. Among them, two key metabolites of paracetamol degradation 4-aminophenol and hydroquinone were identified. On the basis of the obtained results, the authors proposed two different degradation pathway in HJ1012 strain: (1) the initial hydroxylation of paracetamol which results in hydroquinone formation with the release of acetamide and ring opening and (2) the initial decarboxylation of paracetamol to 4-aminophenol, in which amino group is then replaced by the hydroxyl group which results in hydroquinone formation (Fig. 1). The similar catabolic degradation pathways were suggested by Zhang et al. (2013), who described one strain from *Stenotrophomonas* genus (f1 strain) and two *Pseudomonas* strains (f2, fg-2) isolated from aerobic aggregate which use paracetamol as the sole carbon, nitrogen, and energy source. It is worth to note that so far *Stenotrophomonas* genus has not included any known paracetamol degraders. Results obtained by Zhang et al. (2013) showed that the consortium of these three microbial strains was able to degrade paracetamol up to 4 g/L, while pure cultures of f1, f2, and fg-2 strains performed complete degradation of paracetamol at concentrations of 0.4, 2.5, and 2 g/L, respectively. The authors also noticed that the consortium achieved substantially higher degradation rates and significantly better tolerance to paracetamol with a shorter lag time. The cooperative degradation and mineralization capability of microbial consortium was also confirmed by oxygen consumption rate. Intermediates identified during paracetamol degradation by GC-MS technique include 4-aminophenol, hydroquinone, 2-hexenoic acid, succinic acid, malonic acid, oxalic acid, formic acid, nitrate, and nitrite. 4-Aminophenol, hydroquinone, and pyrocatechol were identified as the key degradation metabolites of paracetamol degradation pathway in actinobacteria from *Rhodococcus* genera in studies performed by Ivshina et al. (2006). The oxidative deamination of phenolic intermediates of paracetamol degradation was additionally confirmed by De Gusseme et al. (2011), Wu et al. (2012), and Zhang et al. (2013).

Further degradation of hydroquinone can occur through (1) cleavage of hydroquinone molecule by hydroquinone 1,2-dioxygenase to 4-hydroxymuconic semi-aldehyde and (2) conversion of hydroquinone to 1,2,4-benzenetriol (hydroxyquinol), which may be cleaved by hydroxyquinol 1,2-dioxygenase to maleylacetic acid. The first pathway was proposed for *P. moorei* KB4 strain being able to degrade paracetamol at concentrations as high as 50 mg/L (Żur et al. 2018). The second pathway was proposed, e.g., by Takenaka et al. (2003) for *Burkholderia* sp. AK-5 strain, which utilized 4-aminophenol as the sole carbon, nitrogen, and energy source. Metabolic pathway of 4-aminophenol decomposition by AK-5 strain proceeds via 1,2,4-trihydroxybenzene, 1,4-benzenediol, and maleylacetic acid. Li et al. (2014) described the paracetamol degradation pathway in microorganisms in soil. The proposed mechanism of decomposition of paracetamol is presumably catalyzed by cytochrome P-450 and proceed via hydroxylation to 3-hydroxyacetaminophen, which is then oxygenated to *N*-acetyl-*p*-benzoquinone imine or methylation to *p*-acetanisidide. The first intermediate is further transformed to *para*-benzoquinone. The further conversion of *p*-acetanisidide includes transformation to 4-methoxyphenol and in the next step to the 1,4-dimethoxybenzene. The aromatic structure of the last intermediate is probably cleaved by the ring fission enzymes. This scenario is strongly supported by the presence of 2-hexenoic acid in the soil extract.

**Fig. 2:**
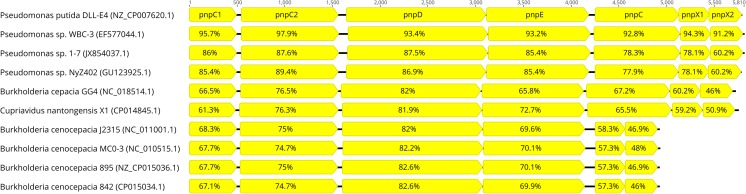
Comparison of *pnpC1C2DECX1X2* gene clusters of ten bacterial strains. Direction of transcription is indicated by arrows. *pnpC1*, small subunit of hydroquinone dioxygenase; *pnpC2*, large subunit of hydroquinone dioxygenase; *pnpD*, 4-hydroxymuconic semi-aldehyde dehydrogenase; *pnpE*, maleylacetic acid reductase; *pnpC*, hydroxyhydroquinone dioxygenase; *pnpX1*, YciI family protein; *pnpX2*, ferredoxin

In the last decades, studies on genetic bases of bacterial degradation of aromatic compounds were focused almost exclusively on aromatic hydrocarbons. Several important plasmids like TOL, NAH, or SAL have been identified. The fact that up to now microbial degradation pathways of naproxen or diclofenac, two the most commonly used polycyclic NSAIDs remains not fully described confirmed the severe gap in this area of knowledge. One of the few genetic experiments concerning nonsteroidal anti-inflammatory drugs and their metabolites was performed by Khan et al. (2006). In this study, *Pseudomonas* sp. ST-4 being able to decompose 4-aminophenol was treated with acridine orange in order to determine the involvement of plasmid-encoded genes in this xenobiotic degradation, since a wide range of ring cleavage enzymes has found to be encoded by plasmids. Indeed, ST-4 strain treated with acridine orange became unable to degrade 4-aminophenol, which confirmed that genes involved in biodegradation were located on plasmid. Several bacterial strains from *Moraxella*, *Pseudomonas*, *Sphingomonas*, *Burkholderia*, *Variovorax*, *Azospirillum*, *Brachymonas*, and *Cupriavidus* genera are capable of utilizing hydroquinone, which may be a product of paracetamol, 4-chlorophenol, 4-fluoro-, 4-bromo-, 4-iodo-, and 4-nitrophenol degradation (Enguita and Leitao 2013). Genes encoding enzymes engaged in degradation of hydroquinone and their regulation were elucidated in the case of 4-nitrophenol-degrading bacterium *Pseudomonas putida* DLL-E4. Nine genes are involved in the degradation of 4-nitrophenol by DLL-E4 strain, specifically *pnpA*, *pnpR*, and *pnpC1C2DECX1X2*, while *pnpC1C2DE* genes are sufficient for transformation of hydroquinone to tricarboxylic acid cycle metabolites. Additional gene *pnpC* encode hydroxyquinol 1,2-dioxygenase, which transform hydroxyquinol to maleylacetic acid further reduced to beta-ketoadipate by maleylacetic acid reductase encoded by *pnpE*. Regulation of genes involved in 4-nitrophenol degradation by DLL-E4 is complex. Supplementation of glucose greatly increases 4-nitrophenol degradation, but inhibits degradation of hydroquinone. Expression of *pnpC1C2DECX1X2* operon is positively regulated by *pnpR* gene, encoding a LysR-type regulator, which also positively regulates its own expression and partially expression of *pnpA* gene that encode 4-nitrophenol 4-monooxygenase (Hu et al. 2014; Chen et al. 2016a, b). In order to better characterize genes involved in hydroquinone degradation, comparative genomics was performed searching in NCBI database using BlastP on default settings with *E*-value cut-off of 1 × 10^−5^. Comparative analysis revealed high conservation of genes order and enabled differentiation of two distinct gene clusters based on the presence of *pnpC* gene that encode hydroxyquinol 1,2-dioxygenase. Lack of this gene was observed in gene cluster of some, but not all bacteria from *Burkholderia* genus. The remaining strains that belong to *Pseudomonas*, *Cupriavidus*, and *Burkholderia* genera possess gene *pnpE* that enables degradation of hydroxyquinol (Fig. 2).

### Ibuprofen

#### Occurrence and toxicity

Ibuprofen (abbreviated as IBU, 2-(4-methylpropyl)phenyl)propanoic acid) currently is the third most popular NSAID worldwide. The annual consumption of ibuprofen in selected EU countries is about 300 t in Germany, 162 t in the UK, and 58 t in Poland (Marchlewicz et al. 2015). In recent years, increasing intake of ibuprofen was reported for Slovakia, Czech Republic, and Finland. Relatively low IBU consumption is observed in Norway and Denmark (Hudec et al. 2012). Ibuprofen is also one of the core drugs in the “Essential Drug List” developed in 2010 by World Health Organization (WHO) (Parolini et al. 2011). Wide prevalence of ibuprofen and its metabolites in sewage and different natural matrices is mainly related to the high therapeutic doses ranging from 600 to 1200 mg/day. Orally administrated ibuprofen at therapeutic doses is rapidly and almost completely (99%) bound to plasma albumin. In human body, up to 15% of ibuprofen is excreted as an unchanged form or glucuronide and thiol conjugates or as metabolites, i.e., hydroxyibuprofen (mostly 2-OH and 3-OH, 1-OH is a minor product), carboxyibuprofen, and carboxyhydratropic acid, which have no apparent pharmacological activity. Nevertheless, conjugates of ibuprofen with glucuronides may be hydrolyzed in the environment (Marchlewicz et al. 2015; Murdoch and Hay 2015). Ibuprofen has been found at different concentrations in environmental samples, e.g., in wastewater from 45 μg/L in Canada (Guerra et al. 2014) to 703–1673 μg/L in Pakistan, 1.38 μg/L in South Africa, and 5.78 μg/L in Belgium (Vergeynst et al. 2015; Ashfaq et al. 2017a), in sludge from 0.009 μg/kg for South Africa (Matongo et al. 2015) to 2053–6064 μg/kg in Pakistan. Luo et al. (2014) reported presence of ibuprofen in WWTPs influents in China, Greece, Korea, Sweden, Switzerland, the UK, and Western Balkan Region (Bosnia and Herzegovina, Croatia and Serbia) at concentration range between 0.004 and 603 μg/L. In soil, ibuprofen concentrations have been detected in the range of 321–610 μg/kg (Ashfaq et al. 2017a) to 0.213 μg/L for soils irrigated with wastewater which contain pharmaceutical residues (Vazquez-Roig et al. 2012). In surface waters ibuprofen has been detected at the median concentration of 0.98 μg/L (Canada), 1.0–67 μg/L (Greece), < 15–414 μg/L (Korea), 5.0–280 μg/L (Taiwan), ND–8.0 μg/L (France), and ND–1417 μg/L (China) (Almeida et al. 2013; Luo et al. 2014). According to Luo et al. (2014), the average concentration of IBU in groundwater for Europe is 3 ng/L, with the maximal concentration of 395 ng/L. Aymerich et al. (2016) reported the concentrations of carboxyibuprofen, 1-hydroxyibuprofen, and 2-hydroxyibuprofen in the WWTP influent as 20.24 ± 7.186, 1.091 ± 814, and 7.768 ± 2.693 ng/L, respectively. In WWTPs sewage containing ibuprofen are mainly treated with AOP, which results in formation of various metabolites with higher or indefinite toxicity compared to the parent compounds, e.g., 1-(4-isobutylphenyl)-1-ethanol, 4-isobutyricbenzaldehyde (Rácz et al. 2011), 2-[4-(1-hydroxyisobutyl)phenyl] propionic acid, 1-ethyl-4-(1-hydroxy)isobutylbenzene, 4-ethylbenzaldehyde, and 4-ethylphenol (Zheng et al. 2011).

Literature data and knowledge about toxicity and influence of ibuprofen on aquatic organisms is still limited. Many authors noticed that most of the studies in this field concern adverse effects at the highest level of the biological hierarchy, thereby omitting the sub-organisms level. The observed changes after acute exposure to IBU in mg/L concentration, which far exceeds the realistic environmental concentrations, may suggest that sub-lethal effects are highly probable. Although the acute toxicity of NSAIDs based on a calculated short-term EC_50_, ranging between 10 and 100 mg/L is rather low, the changes occurring after prolonged exposure to analgesics concerns mainly cyto- and genotoxic effects and imbalance in the oxidative status of cell as well (Parolini and Binelli 2012). Moreover, all further consequences, e.g., growth rate, reproduction, or behavior result always from the alterations on the biochemical level. On the other hand, as many authors highlighted, biochemical alterations and changes in biomarkers endpoints were more strongly expressed in in vivo samples compared to samples tested in in vitro conditions. As Parolini et al. (2011) suggested, living organisms are able to transform parent compound into more toxic intermediates. Such situation was observed by Kayani et al. (2009) for ibuprofen conjugated with diacylglycerol (ibuprofen-DG), where conjugate was responsible for inhibition of cell division and nondisjunction in several pairs of chromosomes. Parolini et al. (2011) demonstrated that chronic exposure to the environmentally relevant concentrations of ibuprofen (0.2, 2.0, and 8.0 μg/L) induced moderate genetic and cellular damage in zebra mussel, *Dreissena polymorpha*, a reference biological model sensitive to various pharmaceuticals, including antibiotics. Imbalances in the activity of enzymes involved in oxidation processes, namely catalase (CAT), superoxide dismutase (SOD), glutathione peroxidase, and the phase II detoxifying enzyme glutathione *S*-transferase (GST), clearly showed that ibuprofen causes oxidative stress. Moreover, authors observed that at the end of exposure to the highest tested concentration (8 μg/L), the activity of CAT, SOD, and GPx return to the initial levels. There are two possible explanations for this state; the balance of the oxidative status was reached or *Dreissena polymorpha* adapted to the changing exposure conditions. Lipid peroxidation and disorders in the same enzymes activity in zebra mussels were also reported by Gonzales-Rey and Bebianno (2012) after 7 days of exposure to ibuprofen. Han et al. (2010) investigated chronic toxicity of ibuprofen for three freshwater species, *Oryzias latipes*, *Daphnia magna*, and *Moina macrocopa* and its influence on hormone balance in in vitro conditions using H295R cell line. Analysis of the results obtained for the cell line revealed that IBU increased production of 17β-estradiol and aromatase activity and decreased testosterone production. Additionally, ibuprofen at concentration of 0.1 μg/L was responsible for a delay in hatching of eggs in *Oryzias latipes*. De Lange et al. (2006) revealed that ibuprofen at concentration ranging from 1 to 100 ng/L decrease activity of amphipod crustacean *Gammarus pulex*. Wang et al. (2016) investigated the influence of IBU at concentrations detected in the environment on expression of three genes involved in detoxification processes in *D. magna*. As shown in the obtained results, the total amount of eggs and the total number of brood per female as well as the body length were significantly decreased after ibuprofen exposure. It was shown that low concentration of IBU (0.5 μg/L) inhibited expression of two of the analyzed genes, while increased value of this (50 μg/L) induced their expression. The third gene expression was inhibited during short-time exposure (6 h), and induced with prolonged time (48 h). The increased activities of CAT, SOD, and GST confirmed occurrence of oxidative stress after IBU exposure. Saravanan et al. (2012) proposed that changes in hemoglobin, hematocrit, mean cellular volume, mean cellular hemoglobin, leukocytes, and plasma glucose and alanine transaminase, which were increased after exposure to ibuprofen (14,200 μg/L, 24 h) in an Indian major carp *Cirrhinus mrigala* may serve as potential biomarkers of toxicity of IBU in organisms in receiving aquatic environments. Alterations of blood parameters and hemocytes after ibuprofen exposure were also reported for *Ruditapes philippinarum* (Aguirre-Martínez et al. 2013) and *D. polymorpha* (Parolini and Binelli 2012). Zanuri et al. (2017) investigated the influence of ibuprofen on successful fertilization of benthos macroinvertebrates, two echinoderms *Asterias rubens* and *Psammechinus miliaris* and one polychaete worm species, *Arenicola marina*. A concentrations of ibuprofen detected in a seawater was reported as 0.01–2317 ng/L. As shown in the obtained results, ibuprofen at these concentrations was responsible for a significant reduction of fertilization success of *P. miliaris*, whereas there were no effects of ibuprofen exposure in oocytes of *A. marina* and *A. rubens*. Toxicity of ibuprofen was also broadly examined against bacteria, mainly using the Microtox test, which enables evaluation of toxic compounds on bioluminescent species, *Allivibrio fischeri*. Di Nica et al. (2017) reported two significantly diverse IC_50_ values after 15 min of ibuprofen exposure, 19.1 and 37.5 mg/L. The negative influence of ibuprofen widely illustrated on both organisms and sub-organism level clearly indicates that the need for monitoring environmental relevant concentrations and total consumption of IBU as well as evaluation of toxicological data are still valid.

#### Biotransformation/biodegradation of ibuprofen

Similarly to paracetamol, despite the environmentally relevant load of ibuprofen, further fate and metabolism of IBU in the natural matrices remain largely unknown. General structure of NSAIDs consist of an acidic moiety (carboxylic acid and enols) attached to a planar, aromatic structure. Some analgesics contain also a polar linking group, which attaches the planar moiety to an additional lipophilic group. Ibuprofen, as diclofenac, naproxen, ketoprofen, and flurbiprofen possess a phenylacetic acid core (PAA). Ibuprofen’s structure, i.e., high branch, the presence of substitutions at the *para* position of the aromatic ring and their spatial configuration suggest its high biodegradation resistance. On the other hand, Parolini et al. (2011) noticed that IBU due to its physico-chemical properties may be characterized by high mobility in the aquatic environments but simultaneously compared to other pharmaceuticals its persistence is lower. It is widely known that cyclic compounds are less susceptible to biodegradation than the aliphatic compounds, analogously, polycyclic aromatic compounds are less vulnerable to degradation than monocyclic one, what is mainly determined by molecular size. Small molecules are favor in their straighter carbon chains and spatially organization, which promote access to enzymes (Musson et al. 2010). To date, only a few examples of bacterial decomposition of ibuprofen have been described.

The first report about the microbial biotransformation of ibuprofen was described by Chen and Rosazza (1994) for *Nocardia* sp. NRRL 5646 strain. Two intermediates formed from the racemic ibuprofen have been identified as ibuprofenol and ibuprofen acetate. Further analysis revealed that the carboxylic acid reductase system responsible for alcoholic derivatives formation is R(-) enantioselective. Anaerobic microbial biodegradation of ibuprofen occur through side chain hydroxylation, which results in carboxyhydratropic acid and ibuprofenol formation. Murdoch and Hay (2005) summarized the metabolism of the most similar to ibuprofen compounds and identified some patterns (i) *meta*-cleavage of cumate (*p*-isopropylbenzoate) molecule occurring in *Pseudomonas putida* F1 strain, where *cmt* operon carrying genes encoding most of the enzymes involved in *p*-cumate catabolism have been identified; (ii) homoprotocatechuate pathway for catabolism of *L*-lysine in *Micrococcus lysodeikticus* and *Bacillus* sp.; (iii) homogentisate degradation pathway of DL-α-phenylhydracrylic, phenylacetic, and 3- and 4-hydroxyphenylacetic acid in *Flavobacterium* strain; (iv) (phenylacetyl)-coenzyme A ligase pathway for Gram-negative bacteria described as the main route of aerobic degradation of PAA acid; (v) *p*-hydroxylation of 2-phenylpropionic acid (2PPA) to 2-(*p*-hydroxyphenyl)propionic acid (HPPA) described in *Streptococcus rimosus*; and (vi) decarboxylation of 2PPA and tropic acid to yield phenylacetaldehyde and subsequent oxidation to phenylacetic acid described in *Pseudomonas cepacia* (Sparnins and Chapman 1976; van den Tweel et al. 1988; Kuge et al. 1991; Andreoni et al. 1992; Eaton 1996; Navarro-Llorens et al. 2005).

**Fig. 3:**
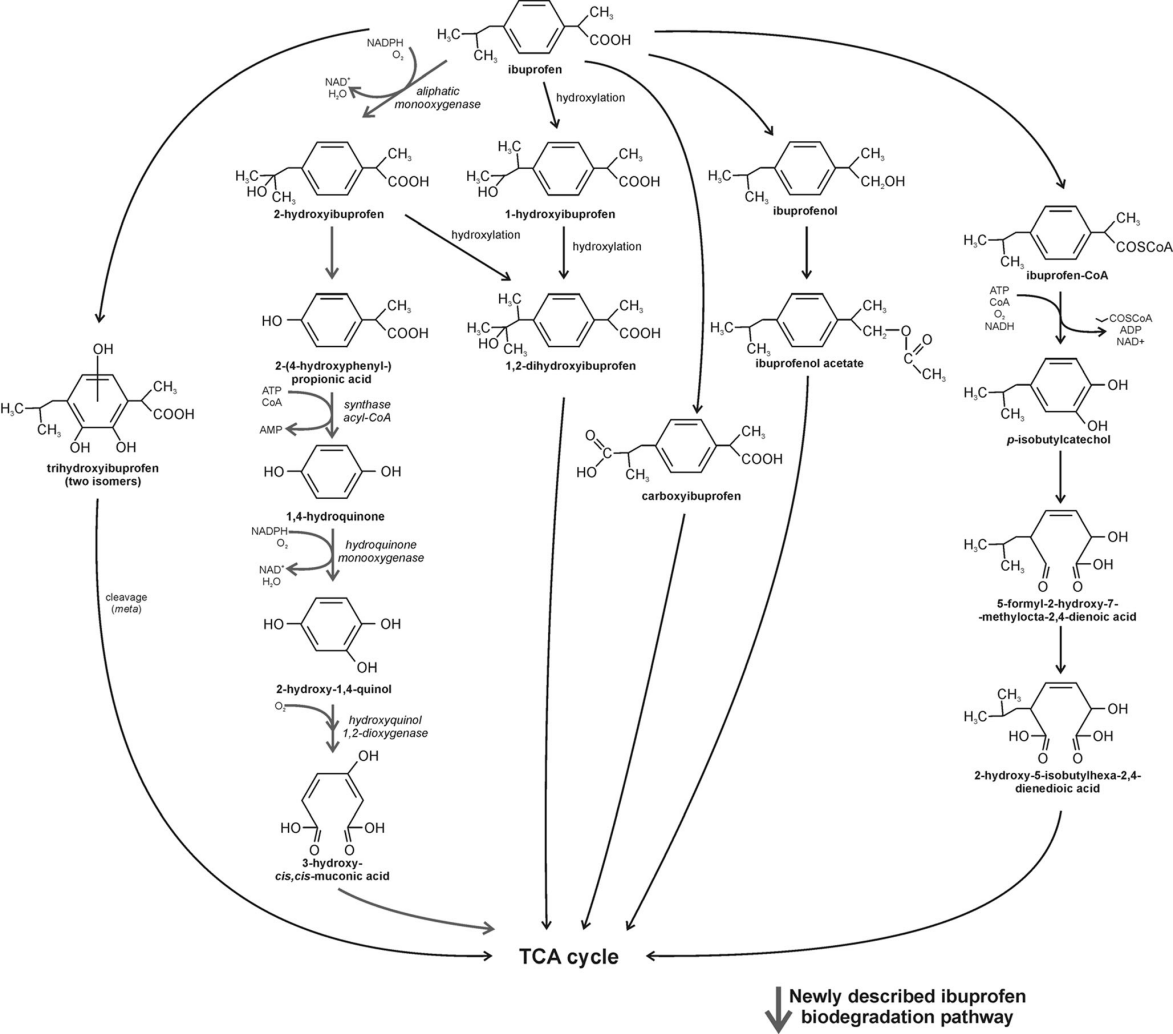
Biodegradation pathway of ibuprofen (Chen and Rosazza 1994; Murdoch and Hay 2005; Quintana et al. 2005; Kagle et al. 2009; Murdoch and Hay 2013; Murdoch and Hay 2015; Marchlewicz et al. 2017b)

**Fig. 4:**
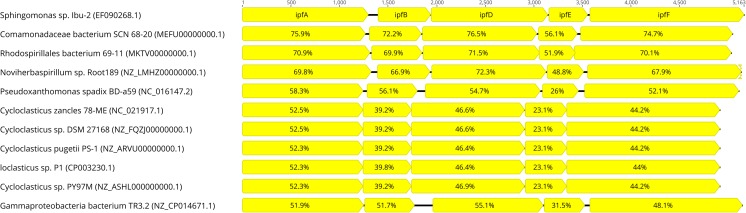
Comparison of *ipfABDEF* gene clusters of 11 bacterial strains. Direction of transcription was indicated by arrows. *ipfA*, large subunit of aromatic ring dioxygenase; *ipfB*, small subunit of aromatic ring dioxygenase; *ipfD*, sterol carrier protein X thiolase; *ifpE*, function unknown; *ipfF*, ibuprofen-CoA ligase

Besides studies performed by Chen and Rosazzaall attempts to describe the bacterial catabolic pathway of ibuprofen had to be based on the studies performed by Murdoch and Hay (2005, 2013). In these studies, the mechanism of microbiological biodegradation of IBU was revealed on the basis of *Sphingomonas* sp. Ibu-2 strain isolated from wastewater treatment plant able to use both enantiomers of ibuprofen as the sole carbon and energy source under aerobic conditions (Fig. 3). Since Ibu-2 is able to remove the propionic acid chain from ibuprofen and other PAA arylacetic acids, i.e., 2-phenylpropionic acid, 3- and 4-tolylacetic acids, and 2-(4-tolyl)propionic acid, to yield catechols or methylocatechols, extensive biochemical and genetic studies on the mechanism of this have been performed. In order to reveal the mechanism of deacylation activity and metabolism of IBU and other PAA compounds, a fosmid library of Ibu-2 total DNA was constructed. Screening the chromosomal library of Ibu-2 DNA in *Escherichia coli* EPI300 allowed to identify one fosmid clone (pFOS3G7) that contained five-gene cluster *ipfABDEF*, involved in transformation of ibuprofen to isobutylcatechol. The *ipfA* and *ipfB* genes putatively encode the two subunits of the aromatic ring of dioxygenase, whereas *ipfD* was identified as a gene encoding sterol carrier protein X thiolase. The *ipfF* gene shows sequence similarity to genes encoding CoA ligases, while for *ipfE* no function was found. Two additional genes, *ipfH* and *ipfI*, were encoding ferredoxin reductase and components of the aromatic dioxygenase system, respectively. On the basis of the genetic and previous biochemical analyses, the authors suggested the following pathway of ibuprofen degradation by Ibu-2 (i) ligation of ibuprofen with CoA by the CoA ligase IpfF, (ii) dihydroxylation of ibuprofen-CoA by the multicomponent oxygenase IpfABHI to yield 1,2-*cis*-diol-2-hydroibuprofen CoA, and (iii) removal of acyl-CoA group by IpfD and IpfE to yield 4-isobutylcatechol (Murdoch and Hay 2005; Kagle et al. 2009; Murdoch And Hay 2013). To expand knowledge in aspect of genetic background of ibuprofen degradation, we have performed a BlastP search against NCBI database using protein sequences of the genes from cluster *ipfABDEF* on default settings with E-value cut-off of 1 × 10^−5^. Based on protein sequence similarity and genes arrangement in cluster we identified *ipfABDEF* cluster in genomes of ten strains with *Sphingomonas* sp. Ibu-2 as query (Fig. 4). Five of the found strains belong to the *Cycloclasticus* genus, namely *Cycloclasticus zancles* 78-ME, *Cycloclasticus* sp. DSM 27168, *Cycloclasticus pugetii* PS-1, *Cycloclasticus* sp. P1, and *Cycloclasticus* sp. PY97M, and are known for its wide aerobic degradation abilities of polycyclic aromatic hydrocarbon (PAH) compounds like naphthalene, phenanthrene, and pyrene. The bacteria of that genus are predominant PAH-degrading bacteria in marine sediments, seawater, and tar (Wang et al. 2008; Messina et al. 2016). The remaining bacteria with *ipfABDEF* cluster include *Pseudoxanthomonas spadix* BD-a59, *Rhodospirillales bacterium* 69-11, *Comamonadaceae bacterium* SCN 68-20, *Noviherbaspirillum* sp. Root189, and Gammaproteobacteria bacterium TR3.2 which were isolated from gasoline-contaminated sediment, ammonium sulfate bioreactor, contaminated soil, thiocyanate bioreactor, and root of *Arabidopsis thaliana*, respectively (Choi et al. 2013; Wattam et al. 2017). Strain BD-a59 has the ability to degrade benzene, toluene, ethylbenzene, and *o*-, *m*-, and *p*-xylene, in turn Gammaproteobacteria bacterium TR3.2 has the potential in degradation of PAH similarly to the strain PY97M which exhibit ability to degrade pyrene and fluoranthene (Choi et al. 2013; Cui et al. 2013; Singleton et al. 2016). The presence of *ipfABDEF* cluster in the genomes of these strains suggests their ability to degrade ibuprofen among other cyclic compounds.

Almeida et al. (2013) described ibuprofen-degrading strain isolated from activated sludge classified as Gram positive and assigned to *Patulibacter* genera. In this study, the authors investigated the biodegradation of ibuprofen in minimal M9 medium and OD-2 broth and the cellular response of bacterium to ibuprofen using quantitative proteomics with the use of a metabolic labeling strategy. Performed analysis revealed a number of proteins which were upregulated even at low concentrations of ibuprofen, i.e., acyl-CoA synthetase, one protein with a Rieske (2Fe-2S) iron-sulfur cluster, which is known to be involved in the initial oxidation of aromatic ring and enoyl-CoA hydratase, a well-known enzyme catalyzing the hydroxylation of double bonds after ring fission. Several ABC transporter proteins involved in the uptake of the particular compounds were also upregulated in response to tested ibuprofen concentrations. Upregulation of ABC transporter proteins, as well as cytochromes and NADH-dehydrogenases, have been also showed for *Corynebacterium glutamicum* and *Pseudomonas* sp. KT2440 cells cultivated on benzoate (Haussmann et al. 2009; Yun et al. 2011), *Pseudomonas* sp. KT244 grown on phenol (Roma-Rodrigues et al. 2010), and *Pseudomonas* grown in the presence of 4-chlorophenol (Cao and Loh 2009). Biotransformation of ibuprofen to trihydroxyibuprofen by the *meta*-cleavage pathway has also been showed for *Variovorax* sp. Ibu-1 strain isolated from activated sludge by Murdoch and Hay (Murdoch and Hay 2015). While previously characterized degradation *ipf* pathway for Ibu-2 strain proceeds via coenzyme A ligation with subsequent dioxygenation and deacetylation steps, which results in isobutylcatechol formation, in *Variovorax* sp. Ibu-1 strain the aromatic ring of ibuprofen undergoes direct trihydroxylates to the product, which is further utilize as ring cleavage substrate. The addition with 3-fluorocatechol, a well-known *meta*-ring fission enzymes pathway inhibitor, demonstrated that poly-hydroxylated metabolites of ibuprofen were only detected when inhibitor was added. It is worth to note that this was a first study with the use of poison 3-fluorocatechol, which enables accumulation of short-lived catecholic intermediates. The same metabolites have been identified in sewage sludge contaminated with ibuprofen, which may suggest that *meta*-ring fission is the most environmentally relevant catabolic pathway of decomposition of ibuprofen (Toyama 2010; Murdoch and Hay 2015). Moreover, the authors suggest that trihydroxylated derivatives of ibuprofen may serves as a dead-end metabolite. It is worth to note that hydroxylated and carboxylated derivatives of ibuprofen, which are characterized by higher toxicity than the parent compound, have been identified in a number of laboratory experiments using both ligninolytic fungi and bacteria as well as physico-chemical processes in WWTPs (Zwiener et al. 2002; Quintana et al. 2005; Murdoch and Hay 2015).

Recently, Marchlewicz et al. (2017b) described a novel ibuprofen degradation pathway occurring in the Gram-positive strain, *Bacillus thuringiensis* B1(2015b) (Fig. 3). High activity of aliphatic monooxygenases as well as phenol and hydroquinone monooxygenases confirmed hydroxylation of both the aromatic ring and aliphatic chain of IBU. GC-MS analysis revealed the formation of several intermediates, i.e., 2-hydroxyibuprofen, 2-(4-hydroxyphenyl)-propionic acid, 1,4-hydroquinone, and 2-hydroxyquinol. 1,4-Hydroquinone as a product of acyl-CoA synthase may be further transform by hydroquinone monooxygenase to 2-hydroxy-1,4-quinol, which is favorable bind by hydroxyquinol 1,2-dioxygenase, an enzyme involved in *ortho* cleavage of aromatic ring, which results in 3-hydroxy-cis,cis-muconic acid formation. Presumably, the final product of ibuprofen degradation is further incorporated in central metabolism. It is clear that for all described studies on the degradation pathways, additional analyses at environmentally relevant concentrations should be performed in order to verify if such biochemical pathways are similar to these observed for artificially high concentrations used in a laboratory scale. It is also worth to mention that Marchlewicz et al. (2017b) observed interesting relationship between removal of ibuprofen and pH medium value. The highest removal efficiency was observed at pH 7.2, whereas in a slightly higher pH (8.0), significant inhibition of ibuprofen biodegradation was observed. Possible explanation concerns a functional state of proteins involved in a decomposition of ibuprofen, since at the pH higher than 6.5, IBU occurs in an anionic form (pK_a_ 4.3). This excludes the hypothesis about the low electrostatic interaction between molecules and binding sites present on the biomass surface. At low and high pH value (suboptimal conditions), the growth of microorganisms is significantly altered (low biomass is related with low degradation capabilities). pH value also affects the activity of degradation enzymes. For example, in B1 strain at pH 8.0, two enzymes engaged in ibuprofen degradation, phenol monooxygenase, and catechol 1,2-dioxygenase showed their maximal activity. The lower activity of hydroquinone monooxygenases at pH 8.0 was probably connected with the loss of the FAD molecule, which is observed for this molecule above pH 7.5. The biodegradation of ibuprofen was examined also in the presence of various co-contaminants. As shown in the obtained results, degradation of ibuprofen by *Bacillus thuringiensis* B1(2015b) strain was enhanced in the presence of phenol, benzoate, and 2-chlorophenol. Simultaneously, removal of phenol and benzoate was also observed. On the other hand, introduction of 4-chlorophenol into the culture completely inhibited degradation of ibuprofen (Marchlewicz et al. 2017a, b).

## Potential application of enzymes involved in NSAIDs degradation in bioremediation and industry

Degradation strategies of aromatic compounds involve hydroxylation catalyzed by monooxygenases or hydroxylation dioxygenases and subsequent aromatic ring fission. Hydroxylation results in several key intermediates formation such as catechol, protocatechuic acid, gentisic acid, or hydroquinol, which are substrates for oxidative ring cleavage catalyzed by dioxygenases. Two families of dioxygenases, intradiol which catalyzes *ortho* cleavage and extradiol which catalyzes *meta*-cleavage, diversified in terms of structure and mechanics, can be distinguished (Melo et al. 2010; Guzik et al. 2013b). Enzymes involved in biotransformation and/or biodegradation of aromatic compounds, including NSAIDs, may be applied in bioremediation and industrial processes due to their resistance to various inhibitors, i.e., metal ions, organic solvents, phenols, or hydrocarbons. To date, direct application of extracted enzymes in the catalytic environmental processes has been limited mainly due to the significant decrease in enzymes activity and potential poisoning or blockage of the enzymes active site. Strategies which allow improving enzymes stability and properties include medium engineering, cross-linking with chemical compounds, protein engineering, or immobilization. Since products of *ortho* ring fission are widely applicable as substrates for chemical synthesis, their role in industry seems to be essential. Currently, one of the major scopes in the field of catalysis is to develop low-cost systems using suitable reusable catalysts that are permanently immobilized on a solid support and are able to perform different cycles of catalysis. Di Nardo et al. (2009) immobilized catechol 1,2-dioxygenase from *Acinetobacter radioresistens* S13 strain able to convert catechol to *cis*,*cis*-muconate (*cc*MA) and subsequent adipic acid on β-cyclodextrins cross-linked with carbonate nanosponges. As shown in the results, the immobilization modified the activity profile of enzyme mainly changes of the optimal pH and temperatures. Compared to the free enzyme thermostability and residual activity of the immobilized enzyme was significantly increased. The usefulness of catechol 1,2-dioxygenase in adipic acid production has also been shown by Guzik et al. (2013a). In this study, a highly active enzyme being able to produce methyl derivatives of *cis*,*cis*-muconic acid was isolated from the environmental strain *Stenotrophomonas maltophilia* KB2. A *catA* gene encoding catechol 1,2-dioxygenase was identified in strains able to convert *cis*,*cis*-muconic acid and was used to produce great amounts of this acid by recombinant *Escherichia coli* cells carrying gene from *Pseudomonas putida* mt-2 (Kaneko et al. 2011). Han et al. (2015) reconstructed a new synthetic pathway of *cc*MA production by regulating the constitutive expression of catechol 1,2-dioxygenase from the *Acinetobacter* sp. ADP1 strain. The tenfold greater enzyme activity was possible to achieve due to the mutations in the protein structure. The ring fission by the *ortho* pathway and activity of catechol 1,2-dioxygenase and phenol hydroxylation enzymes were also shown in filamentous fungi from *Fusarium*, *Aspergillus*, *Penicillium*, and *Graphium* genera, which suggest their application in the treatment of phenol contaminated areas (Santos and Linardi 2004). Dos Santos et al. (2009) studied degradation of phenol by immobilized and free *Aspergillus pullulans* FE13 cells. Despite the loss of catechol 1,2-dioxygenase activity, alginate-immobilized cells, after immobilization cells remained viable for a longer period, and increased the efficiency of phenol degradation was observed. Zucolotto et al. (2006) developed a highly sensitive biosensor for detection of catechol derived from pesticides and other industrial wastewater degradation. Chlorocatechol 1,2-dioxygenase (CCD) was immobilized in nanostructured films interleaved with poly(amidoamine) dendrimer in a layer-by-layer manner. The results showed that immobilization did not affect the CCD activity and obtained films were able to detected catechols even at a concentration of 10^−19^ M. Catechol 1,2-dioxygenase was also isolated from *Sphingomonas xenophaga* QYY (Guo et al. 2009) and *Rhodococcus opacus* 1CP (Matera et al. 2010). Dioxygenase isolated from *R. opacus* 1CP and named *Rho* 1,2-CCD was shown to be able to degrade catechols and its methylated and chlorinated derivatives as well as protocatechuate. Chlorocatechol dioxygenases due to the steric and electron distribution in the large active site are found to be capable of utilizing a broad range of aromatic compounds, including methylated derivatives of catechol (Melo et al. 2010). In the study performed by Zhang et al. (2017), protocatechuate 3,4-dioxygenase (P34O), isolated from *Rhizobium* sp. LMB-1 was covalently bound to supermagnetic (3-Aminopropyl) triethoxysilane (3-APTES)-modified Fe_3_O_4_ nanoparticles using the glutaraldehyde method. Immobilization of P34O greatly increased its properties, i.e., thermostability, kinetic parameters, and reusability. Metabolic pathway proceeds via protocatechuate 3,4-dioxygenase ring fission is also used to production of beta-ketoadipic acid and muconolactone from aromatic compounds related to lignin, which are recently useful in production of industrial chemicals such as muconic acid, polyhydroxyalkanoate, or guaiacol (Okamura-Abe et al. 2016). Mycroft et al. (2015) inserted genes encoding protocatechuate 4,5-dioxygenase and protocatechuate 2,3-dioxygenase to aromatic lignin-degrading strain *Rhodococcus jostii* RHA1. It resulted in formation of aromatic pyridine-dicarboxylic acids used for bioplastic synthesis. The presence of microorganisms with high catechol 2,3-dioxygenase activity was detected in oxygen limited environment contaminated with aromatic hydrocarbons (Táncsics et al. 2015). Hydroxyquinol 1,2-dioxygenase is a well-known enzyme involved in chlorobenzene, aminophenols, and nitrophenols degradation. As Guzik et al. (2013b) and Takenaka et al. (2003) noticed during degradation of such components, hydroxyquinol is a key intermediate; thus, enzymes being able to decompose it play the important role in their biodegradation. Recombinant hydroxyquinol 1,2-dioxygenase derived from *Arthrobacter chlorophenolicus* A6 immobilized on single-walled carbon nanotubes using physical adsorption and covalent bounding by Suma et al. (2016) was characterized by increased temperature range and resistance to harsh environmental factors, i.e., ionic strength.

## Conclusions

Despite the widespread use of analgesic paracetamol and NSAID ibuprofen and, consequently, their unintended presence in the environment, the mechanisms of biological degradation and their genetic bases remain poorly understood. Therefore, the need for isolation and characterization of new bacterial strains being able to degrade paracetamol and ibuprofen is still valid. Further research in biodegradation area should be focused on development of highly effective treatment systems, e.g., by immobilization of microorganisms with increased metabolic properties, which will combine the high removal rate and low-cost options. The second most pressing aspect of analgesic degradation concerns the unraveled genetic bases of degradation abilities, e.g., identification of genes, operons, and regulation of their expression. Obtaining mutants, metagenomics studies, sequencing of whole bacterial genomes, and comparative genomics are most often used for those purposes. On the other hand, enzymes responsible for the increased degradation of xenobiotics are often isolated and used for various industrial processes.
